# Scream’s roughness grants privileged access to the brain during sleep

**DOI:** 10.1038/s41598-025-01560-8

**Published:** 2025-05-14

**Authors:** Guillaume Y. T. Legendre, Maëva Moyne, Judith Domínguez-Borràs, Samika Kumar, Virginie Sterpenich, Sophie Schwartz, Luc H. Arnal

**Affiliations:** 1 Department of Basic Neuroscience, University of Geneva, Rue Michel Servet 1, CH-1211 Geneva, Switzerland; 2 Defitech Chair for Clinical Neuroengineering, Neuro-X Institute (INX) and Brain Mind Institute (BMI), École Polytechnique Fédérale de Lausanne (EPFL), Geneva, Switzerland; 3 Defitech Chair for Clinical Neuroengineering, Neuro-X Institute (INX) and Brain Mind Institute (BMI), École Polytechnique Fédérale de Lausanne (EPFL) Valais, Clinique Romande de Réadaptation Sion, Sion, Switzerland; 4 Department of Clinical Neuroscience, University of Geneva, 4 rue Gabrielle-Perret-Gentil, Genève 14, CH-1211 Switzerland; 5 Department of Clinical Psychology and Psychobiology, Institute of Neurosciences, University of Barcelona, Barcelona, Spain; 6 Department of Psychology, University of Cambridge, Downing Street, Cambridge, CB2 3EB UK; 7 Swiss Center for Affective Sciences, University of Geneva, chemin des mines 9, Geneva, CH-1202 Switzerland; 8 Université Paris Cité, Institut Pasteur, AP-HP, INSERM, CNRS, Fondation Pour l’Audition, Institut de l’Audition, IHU reConnect, Paris, 75012 France

**Keywords:** Sleep, Auditory perception, Emotions, EEG, Non-REM sleep, Sleep, Emotion, Auditory system, Sensory processing

## Abstract

During sleep, recognizing threatening signals is crucial to determine when to wake up and when to continue vital sleep functions. Screaming is perhaps the most efficient way for communicating danger at a distance or in conditions of limited visibility. Screams are characterized by rapid modulations of sound pressure in the so-called roughness range (i.e., 30–150 Hz) which are particularly powerful in capturing attention. However, whether these rough sounds are also processed in a privileged manner during sleep is unknown. We tested this hypothesis by presenting human participants with low-intensity vocalizations, including rough screams and neutral, low-roughness vocalizations during wakefulness and during a full night of sleep. We found that screams evoked cortical responses with higher theta phase-consistency as compared to neutral vocalizations during both wakefulness and NREM sleep. In addition, screams boosted sleep spindle power, suggesting elevated stimulus salience. These findings demonstrate that, even at low sound intensity (e.g., from a distant source), vocalizations’ roughness conveys stimulus relevance and enhances exogenous processing in both the waking and sleeping states. Preserved differential neural responses based on stimulus salience may ensure adaptive reactions in a state where the brain is mostly disconnected from external inputs.

**Supplementary Information:**

The online version contains supplementary material available at 10.1038/s41598-025-01560-8.

## Introduction

The ability to process threat cues regardless of current attentional or vigilance state is essential to rapidly react to danger and ultimately ensure survival. Even during sleep, it is crucial to detect threatening signals and potentially return to wakefulness to respond to imminent danger. Some sounds have the capacity to disrupt sleep continuity more than others, especially if they are relevant for the sleeper. For instance, parents wake up more often upon hearing the cry of their own baby as compared to that of other babies^1^. Similarly, hearing one’s own name (as compared to someone else’s name) induces a distinctive EEG response during sleep^2–5^. Other auditory properties carrying meaningful information for the sleeper, such as emotional prosody and familiarity of voices, can be discriminated by the sleeping brain^3,6–9^. This ability is critical for the detection of threats during sleep. Auditory emotional salience processing during sleep has been demonstrated by measuring the number of awakenings^1^, the amplitude of event-related potentials (ERP)^4,8^, EEG power^3,5,6^, and sleep oscillations^2,5,7^. Regarding sleep oscillations, auditory stimuli played below the awakening threshold can trigger slow-waves (i.e. large deflections of the EEG signal between 1 and 4 Hz^10^ and sleep spindles (i.e. waxing and waning oscillation with a peak frequency around 12.5 Hz^10–12^. Evoked slow-waves during NREM sleep are traditionally considered markers of unexpectedness^13^, while spindles may follow both evoked and spontaneous slow-waves^7,11^. Thus, the detection and salience of a stimulus by sleeping participants can be probed via ERP and in the power of typical sleep oscillations such as delta (i.e. 1–4 Hz^10,14^ and sigma frequency range (i.e. 12–15 Hz^10–12,14^.

**Fig. 1:**
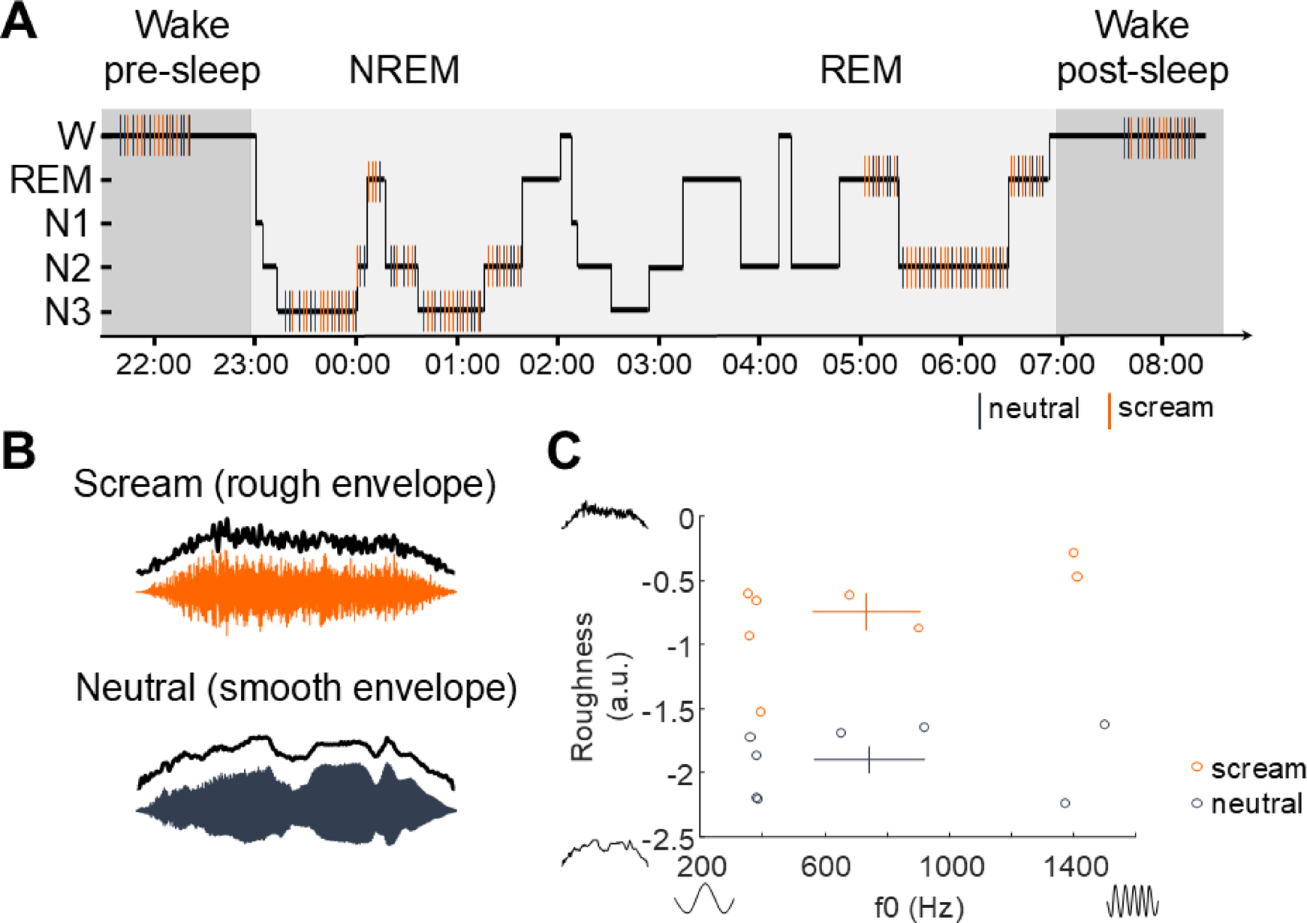
Stimulation protocol and acoustic stimuli characterization. (**A**) Example hypnogram of one participant depicting the presentation of stimuli as a function of sleep stages over one night. Stimuli were first played in a brief session of wakefulness before sleep, during the three first hours of sleep, during the two last hours of sleep and after sleep, during another session of wakefulness. (**B**) Examples of two auditory stimuli waveforms in each condition: neutral (dark gray) and screamed (orange) vocalizations. Overlaid black line visually depicts that neutral voices are smoother than rough screams. (**C**) Eight screams and eight neutral vocalizations (from Arnal et al. 2015) were presented to participants in a pseudo-randomized fashion. This subset of vocalizations was chosen to cover a wide range of fundamental frequencies (f0, paired across neutral and screamed vocalizations) and roughness levels. As quantified in Arnal et al. 2015, the scatter plot reveals that screams and neutral vocalization mainly differ in their roughness levels. Crosses represent the mean (center) and standard error of the mean (tails) of pitch (x-axis) and roughness (y-axis) for screams (orange) and neutral vocalizations (dark gray).

Research focusing on auditory emotional processing during sleep has mostly used complex, natural vocalizations as stimuli. However, whether the emotionality of vocalizations may rely on specific acoustic parameters to trigger more efficient reactions during sleep is unclear. Intensity is likely a determining factor, which informs about the proximity (and size) of a sound’s source, i.e. the closer the louder. However, intensity does not unambiguously convey the relevance of an alarm call, nor may loud sounds leave enough time for an adapted reaction. Other acoustic features –contained, for example, in the alarm vocalizations of conspecifics– may be essential for waking up and responding quickly to a distant threat. Previous works focusing on alarm communication in humans^15^ have shown that screamed vocalizations, unlike emotionally neutral vocalizations, exhibit fast amplitude modulations in the so-called roughness range (i.e. 30–150 Hz; see Fig. 1B and C). This acoustic feature is specifically exploited in natural and artificial alarm signals to trigger more systematic and faster reactions than neutral sounds, even at low signal to noise ratio. Owing to its aversive nature, roughness –unlike pitch or slower speech features, e.g., syllables or prosody– is preserved for alarm signaling to convey threats^15^ or to express the emitter’s intent to be exceptionally salient. A subsequent work studying intracranial responses to synthetic sounds demonstrated that subjective aversion to rough sounds coincides with the widespread, sustained synchronization of brain networks involved in salience and emotional processing^16^. Based on these findings, we hypothesized that, as an intrinsically salient feature, roughness might boost exogenous neural processing of screams across varying vigilance states.

Here, we used surface electroencephalographic (EEG) recordings to study brain responses to human alarm vocalizations (screams) as a function of vigilance states. We presented participants with low-intensity vocalizations during wakefulness (before and after one night of sleep, see Fig. 1A) and during NREM sleep (during a full night of sleep). To test the hypothesis that roughness – the specific acoustic feature of screams– is processed in a privileged manner during sleep, we contrasted the effects of screamed (rough) and neutral (non-screamed) vocalizations (Fig. 1B). We used a set of vocalizations (from Arnal et al.^15^ consisting in screamed and neutral meaningless utterances, matched paired in pitch, emitted by four female and four male speakers. To test the effects of screams’ specific features on brain responses we carefully controlled that the vocalizations spanned only two main dimensions: roughness and pitch (Fig. 1C), all other parameters (intensity, acoustic envelope attack and offset) being equal.

Our central hypothesis was that (rough) screams would induce a fundamental and qualitative change in neuronal message-passing to enhance salience-related responses such as slow-waves and sleep spindles during sleep. We contrasted brain responses to screams and neutral vocalizations as a function of two main vigilance states: wakefulness and NREM sleep. We assessed these effects using classical ERP and spectrally resolved analyses in typical frequency bands such as the delta and sigma. Recent findings showed that the detection of an auditory target and the recruitment of attentional processes elicit increased ITPC of cortical responses in the delta^17,18^ and theta (4–8 Hz) range^18–20^, which indicates an enhanced response consistency across trials. Considering this, we sought to test whether roughness enhanced the ITPC of auditory responses. To exclude potential confounds from evoked slow waves, we considered a frequency range unaffected by slow waves (i.e. the theta range) for ITPC analyses.

## Methods

### Participants

Twenty-three healthy participants were recruited via posters displayed at the University of Geneva. Participants reported normal audition, no known neurological or psychiatric disorder, and no drug consumption. This experimental protocol was approved by the local ethical committee for research (“Commission cantonale d’éthique de la recherche de Genève”) and all research was performed in accordance with the relevant guidelines and regulations of the local ethical committee. All participants signed an informed consent validated by the local ethical committee. All participants were blind to the experimental conditions but were informed that sensory stimulations would be played during their sleep. The data from six participants were excluded from the analyses, due to poor EEG signal (*N* = 3) or to bad sleep quality (*N* = 3). The data from two other participants were rejected from the analysis due to insufficient number of unartefacted trials (< 100 trials) in wakefulness or NREM sleep. Due to the limited number of trials in REM sleep, we did not investigate this sleep stage. The final sample includes 15 participants (9 females; age range = 21.87 ± 1.73 - mean ± standard deviation). For more information about participant demographics, see our previous paper^9^.

### Stimuli

Two distinct sets of vocalizations were played to the participants during wakefulness and during one night of sleep. The first set consisted of pseudowords and the ensuing results have already been reported elsewhere^9^. The second set of stimuli was used in the current experiment and consisted of neutral and screamed meaningless (e.g. [‘Aaaah’]) vocalizations detailed in Arnal et al.^15^. The vocalizations were recorded in a soundproof room with a microphone (SM57, Shure Inc., Niles, IL) placed at a 2 m distance from the speaker. 8 human speakers (4 females) were asked to produce 4 meaningless screamed vocalizations and 4 meaningless loud neutral vocalizations. The speakers were explicitly asked not to scream the loud vocalizations (i.e. use no roughness in their voice). We selected a subset of eight screams and eight neutral vocalizations from an equal number of male and female speakers, ensuring maximum variability within the roughness and pitch dimensions. To avoid spurious effects due to fundamental frequency (F0) differences between neutral and screamed vocalizations, we matched the F0 of each neutral vocalization with the scream’s F0 of the same speaker using a voice pitching procedure as implemented in Praat (http://www.praat.org/). All vocalizations were acquired at 44.1 kHz and subsequently resampled at 16 kHz. All vocalizations were edited to last 750 ms and normalized for sound attacks using onset and offset sine ramping of 100 ms and for intensity (using RMS normalization). The roughness values of each vocalization (Fig. 1B and C) was estimated using the Modulation Power Spectrum procedure^21^. Briefly, a 2D Fourier transform was applied on the spectrogram of each sound to extract the modulation power spectrum and the power modulations within the 30–150 Hz frequency range were averaged to get a single roughness measure per vocalization. On average screamed vocalizations had a roughness of −0.74 ± 0.38 a.u. (mean ± SD) and a fundamental frequency at 733 ± 457 Hz and neutral vocalizations a roughness of −1.89 ± 0.27 a.u. and a fundamental frequency at 743 ± 470 Hz. Additional details regarding the sound editing procedures and acoustic characterization of these vocal excerpts can be found in Arnal et al.^15^.

### Experimental procedure

#### Sound calibration

The intensity of sound presentation was adjusted individually according to the following procedure. Participants were asked to sit comfortably in a chair in a soundproof room, with in-ear earphones (Sennheiser CX 3.00) and to listen to several presentations of the same sound to determine their hearing threshold. Participants were asked to report when the sound was played by pressing a key. Sounds were played with a random temporal jitter ranging from 3 to 5 s. The volume of the sound was set using a self-adjustment procedure increasing twice the volume up to self-reported hearing threshold (starting from − 90 digital dB with steps of +/-1.5 digital dB) and decreasing twice the volume down to self-reported hearing threshold (starting from − 10 digital dB with steps of +/-1.5 digital dB). The auditory threshold was set as the averaged volume across the four adjustments. The volume of stimulus presentation was then set at 130% of the auditory threshold (for both awake and asleep stimulation).

#### Polysomnographic recording

EEG was recorded with cup electrodes set on Fpz, F3, Fz, F4, T3, C3, Cz, C4, T4, P3, Pz, P4 and Oz positions of the international 10–20 system, and plugged to a 16-electrode EEG amplifier (VAmp model; BrainProducts GmbH). Electrodes were stuck with an adhesive and conductive paste (EC2; Cadwell Industries, Inc.). Additionally, two electro-oculogram (EOG) electrodes were placed one centimeter above and right to the external right eye canthus and another one centimeter below and left to the external left eye canthus of the participant according to sleep scoring guidelines^22^. Finally, two electro-myogram (EMG) electrodes were placed on the chin, one centimeter to the right and to the left of the dimple of the participant. Electrical signal was recorded at 500 Hz and referenced online to the Fpz electrode. EEG data were filtered online using highpass filter at 0.1 Hz and lowpass filter at 250 Hz.

#### Stimulation protocol

 Once the EEG was set up, the participant sat comfortably on a chair in front of a computer, and was asked to stay awake, eyes closed, without moving for one hour, while listening to the two sets of sounds (Fig. 1A). To keep participants fully awake, the experimenters entered the room to check on the participant and explain the instructions once more every 15 min or when signs of drowsiness were detected. The sounds were played one after another with a random jitter of 5 to 11 s (drawn from a uniform distribution with a millisecond precision) in alternating blocks of one set of sound or the other. Each block comprised 22 stimuli and lasted about 219 s. Once the awake session was completed, the participant was invited to go to bed and fall asleep for the night. The polysomnographic recording of the participant was monitored online by the experimenters. Overnight stimulation started as soon as the first period of N3 sleep was observed by the experimenter. It was stopped if the participant awoke and resumed when the participant went back to stable (N2, N3 or REM) sleep. To collect comparable amounts of brain responses in wakefulness, NREM and REM sleep (the later not analyzed due to insufficient number of trials per participant), each participant was stimulated during the first ~ 3 h of the night - rich in NREM sleep - and during the last expected ~ 2 h of sleep (estimated from self-reported sleep schedule) - rich in REM sleep. After the night, when the participant was fully awake and ready, a final stimulation session of about 1 h was performed during wakefulness. Data collected in wakefulness pre- and post-sleep were pulled together for the analysis or evoked responses during wakefulness.

### Sleep scoring

Sleep was scored by 2 experienced scorers who visually classified the polysomnographic signal (consisting of EEG, EOG and EMG signal) on continuous 20-second epochs according to standards of sleep scoring^10^. For a detailed summary of sleep measures, see our previous paper^9^.

### EEG preprocessing

EEG signal was first epoched from − 1 to 2 s relative to stimuli onset. Epochs were labeled as wakefulness if the stimulus onset occurred in a 20 s-window of wakefulness, as NREM sleep if the stimulus onset occurred in a 20 s-window of N2 or N3, and as REM sleep if the stimulus onset was in a 20 s-window of REM sleep. Each epoch was then visually inspected and rejected if noisy. Epochs overlapping with micro-arousals (based on manual sleep scoring) were rejected. Channels were then visually inspected and noisy channels were rejected. The electrodes were re-referenced to the common average reference.

### Event-related potentials

Preprocessed continuous signal was bandpass filtered between 0.1 and 30 Hz with a 1st order butterworth filter and was re-segmented into epochs from − 1 to 2 s relative to stimuli onsets. Epochs were baseline corrected using a 500 ms pre-stimulus window. To observe the difference of brain response to screams and neutral vocalizations within the theta frequency range (Fig. S1), we further filtered the epochs between 4 and 8 Hz with a 4th order zero-phase butterworth filter. This last filter was only applied to the data of figure S1.

### Time-frequency analysis

A time-frequency decomposition was carried on continuous preprocessed EEG data. The signal was decomposed into 30 frequency bins, from 1 to 30 Hz with steps of 1 Hz, with Morlet wavelets convolution. Wavelets had a Full Width at Half Maximum (FWHM) linearly decreasing from 1 s to 0.33 s (corresponding to 1 cycle for the 1 Hz wavelet and 10 cycles for the 30 Hz wavelet), following the method recommended by Cohen (2019)^23^. Then, the signal was epoched from − 2 to 3 s relative to stimuli onset and downsampled by a factor of 10. To compute power changes, the modulus of the complex number was squared. We then took the natural logarithm of this number and multiplied it by 10 (decibel transformation). Finally, a baseline correction was applied to each time series (frequency and trial wise) by subtracting the mean power between − 0.5 and 0 s relative to stimulus onset. To compute inter-trial phase coherence (ITPC), the argument function was applied to complex numbers extracted by wavelet convolution to obtain the phase in radian and the following formula was applied:

$$\:{ITPC}_{f,t}\:=\frac{1}{N}\left|{\sum\:}_{k=1}^{N}{e}^{i{\phi\:}_{f,t}^{k}}\right|\:$$

With N the number of trials and $\:{{\upphi\:}}_{\text{f},\text{t}}^{k}$ the phase at trial k, at frequency f and time t. Time series (frequency and participant wise) were then corrected by subtracting the average ITPC between − 1 and 0 s relative to stimulus onset. To compare the time course of frequency-decomposed responses between conditions, the power was averaged within canonical frequency bands of sleep research: delta (1–4 Hz^10,14^, theta (4–8 Hz^10,14^ and sigma (12–15 Hz^10–12,14^. For visualization purposes, time-frequency heatmaps of power, ITPC and power modulations related to sound features (pitch and roughness) were upsampled by a factor of 4 in time and frequency by means of cubic spline interpolation. This upsampling procedure was not applied to line plots and statistics. Colormaps used for heatmaps and topographical representations were obtained from the matlab implementation of cmocean^24^.

### Statistics

First-order statistics were computed by averaging trials within units of observation (participant), stimulus type and sleep stage. Exception was made for ITPC, which was calculated on several trials. In this case, the ITPC was calculated for each participant and condition and the result was considered as the first-order statistic. A second exception was made to investigate the linear relation between sigma power and acoustic features. For each participant and in each sleep stage, all stimuli (neutral vocalizations and screams) were included. At each time-point, each frequency and each electrode, the power values across trials were regressed with z-scored roughness and with z-scored pitch values of stimuli. Then, the slope of the regression (beta values) was used as first-order statistics for each participant. Power, ITPC and regressions were computed for each channel separately and all channels were then averaged together within each participant. Finally, second order statistics and p-values were computed across units of observation. Time courses of the paired difference between responses to screams and neutral vocalizations, and influence of roughness and pitch on time courses of the response to all types of vocalizations were statistically tested within a window of 0 to 2 s post stimulus onset for power analyses and power regression analyses and within a window of 0 to 1 s for ERP and ITPC analyses using cluster permutation as evoked time-locked activities were shorter than induced activities. Clusters were selected as consecutive points whose p-values were below 0.05 (_thr_=0.05) as tested with t-tests. Then, cluster values were defined as the sum of the t-values within the cluster (noted as t_cluster_ in the text). 5000 Monte Carlo simulations (*N* = 5000) were performed to compute the distribution of the cluster values across permutations. The p-value of the cluster computed on the distribution of the cluster values is reported in the text as p_cluster_. Statistical testing based on cluster permutation was performed with routines from the Fieldtrip toolbox^25^. The original values of power, ITPC, or power gain were averaged across time points of significant clusters; accordingly, the mean across participants and the standard deviation are reported in the text for each significant cluster. In addition, we estimated the effect size of the average values within significant clusters by computing the Cohen’s d as:

$$\:d\:=\:\frac{\mu\:}{s}$$

where µ is the above-mentioned mean across participants and s is the above-mentioned standard deviation across participants.

## Results

### Brain responses to screamed and neutral vocalizations during wakefulness

**Fig. 2:**
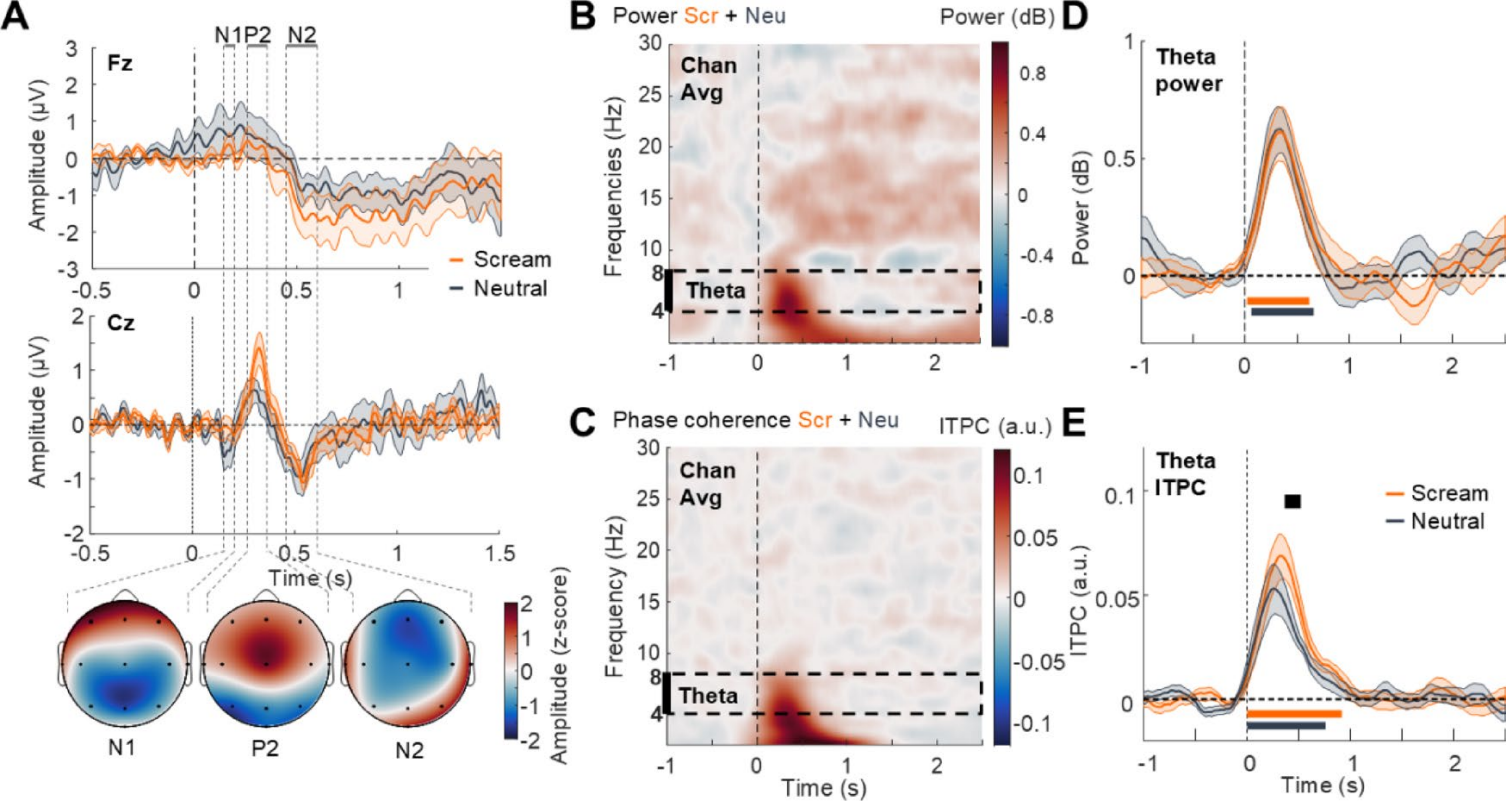
EEG responses to vocalizations during wakefulness. (**A**) Screams (orange) and neutral vocalizations (gray) evoke typical N1, P2 and N2 ERP components on Fz (top panel), and more prominent responses on Cz (middle panel, and related topographies below). P2 magnitude appears larger for screams than neutral vocalizations although this difference does not resist correction for multiple comparisons. (**B**, **C**) The power and ITPC (respectively B and C) time-frequency map of the average responses across all electrodes indicates that vocalizations evoke typical waves in the delta-theta (1–8 Hz) range. To analyze consistently wakefulness and sleep data, we excluded the delta (1–4 Hz) frequency range that could potentially be influenced by sleep oscillations (i.e., slow waves). The dashed black rectangle indicates the theta (4–8 Hz) frequency range investigated in further analyses. (**D**) Power response was averaged in the theta frequency range for screams (orange trace) and for neutral vocalizations (gray trace) but no measurable difference of power between them was observed. (**E**) ITPC response was also averaged in the theta range and shows that auditory phase-locked responses are larger for screams (orange trace) than for neutral vocalizations (gray trace). Shaded surfaces in plots indicate SEM. Significant statistical differences (corrected using cluster-based permutations) are plotted as thick horizontal lines (orange for screams against 0; gray for neutral vocalizations against 0 and black for the difference screams vs. neutral vocalizations).

We first measured ERPs to screamed and neutral vocalizations. Although stimuli were presented at low sound intensity, auditory ERPs exhibited typical N1-P2-N2 components^26^ on Cz (Fig. 2A) with expected centro-parietal, central, and fronto-central topographies, respectively. A qualitative comparison suggested that the P2 component was larger for screams than for neutral vocalizations although this difference did not survive cluster-based correction for multiple comparisons (Fig. 2A; No cluster was found between 0 and 1 s post-stimulus onset on Fz and cluster with the lowest p-value on Cz from 0.312 to 0.352 s; mean voltage difference ± SD (microV): 8.24e-01 ± 1.11, *N* = 15, t_cluster_=57.888, p_cluster_=0.077, d = 0.74).

We then used spectrally resolved analyses to investigate potential differences in power or intertrial phase coherence (ITPC) across single-trial EEG responses to screamed and neutral vocalizations. The time-frequency map of averaged responses indicates increased power (Fig. 2B) and ITPC (Fig. 2C) in the theta and delta frequency ranges over the time of stimulation. Focusing on auditory responses in the theta frequency-range^17,18^, we found that both screamed and neutral vocalizations induced a power increase (screams: significant cluster from 0.020 to 0.620 s, mean power difference ± SD (dB): 4.10e-01 ± 3.19e-01, *N* = 15, t_cluster_=133.89, p_cluster_=0.001, d = 1.29; neutral: significant cluster from 0.060 to 0.660 s, mean power difference ± SD (dB): 4.19e-01 ± 2.87e-01, *N* = 15, t_cluster_=141.41, p_cluster_=0.001, d = 1.46), with no significant difference between the two conditions (Fig. 2D; Cluster with the lowest p-value from 1.540 to 1.740 s; mean power difference ± SD (dB): -2.09e-01 ± 2.80e-01, *N* = 15, t_cluster_=-30.69, p_cluster_=0.075, d = 0.74). The evoked increase in theta ITPC was significant for screamed (Fig. 2E; significant cluster from 0 to 0.920 s; mean ITPC difference ± SD (a.u.): 3.60e-02 ± 1.76e-02, *N* = 15, t_cluster_=257.85, p_cluster_<0.001, d = 2.04) and for neutral vocalizations (significant cluster from 0 to 0.760 s; mean ITPC difference ± SD (a.u.): 3.14e-02 ± 2.34e-02, *N* = 15, t_cluster_=177.67, p_cluster_<0.001, d = 1.34). We also found a larger increase of ITPC for screamed compared to neutral vocalizations between 0.360 and 0.520 s post-stimulus onset (Fig. 2E; significant cluster from 0.360 to 0.560 s; mean ITPC difference ± SD: 2.07e-02 ± 2.68e-02, *N* = 15, t_cluster_=25.69, p_cluster_=0.048, d = 0.77), suggesting that screams’ roughness enhanced the phase-consistency of the evoked responses in the theta range. Consequentially, when filtering individual ERPs within the theta range, the response to screams looks more similar across participants as compared to neutral vocalizations (Fig. S1, upper panels).

### Brain responses to screamed and neutral vocalization during NREM sleep

**Fig. 3:**
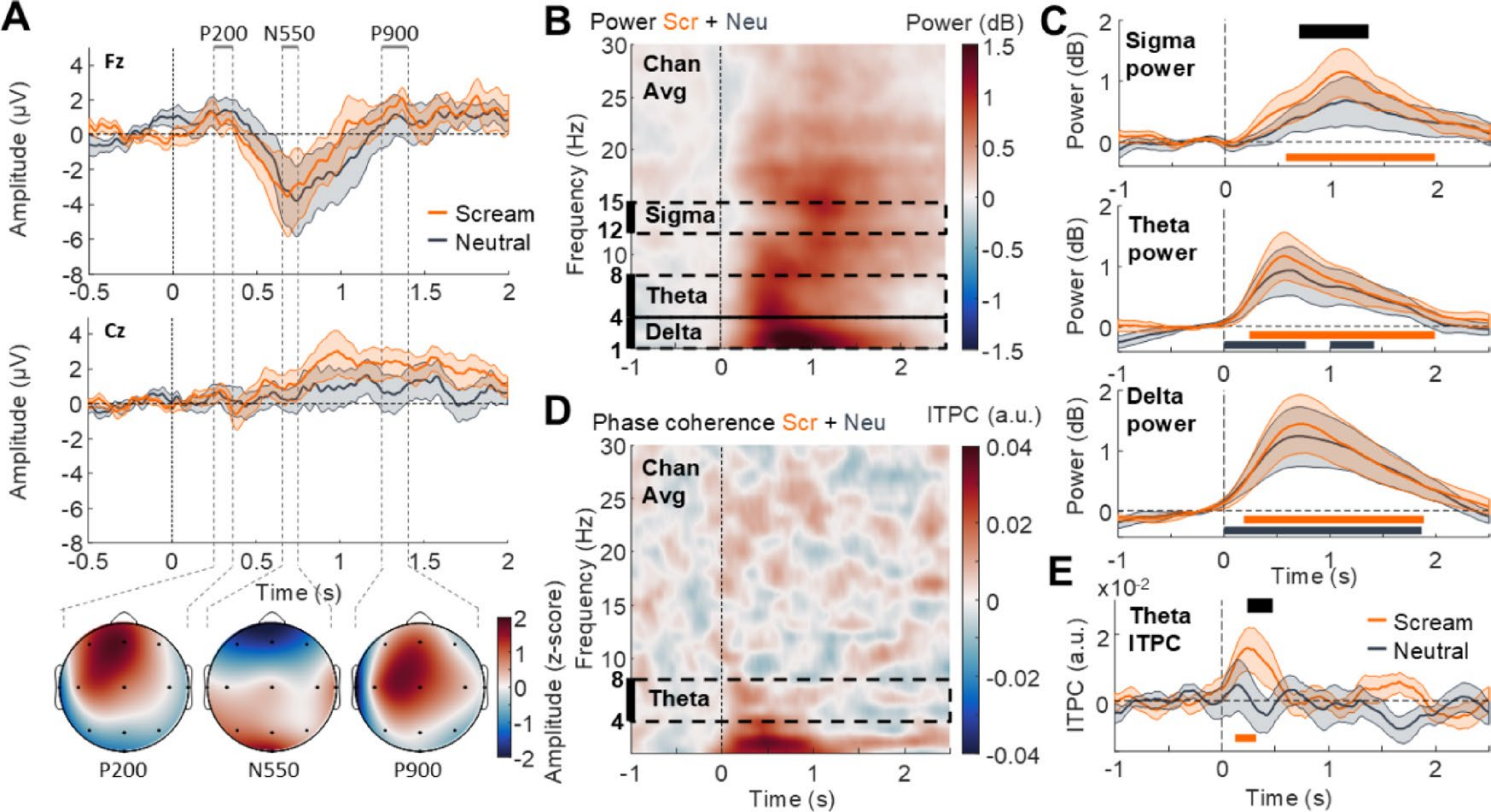
EEG responses to vocalizations during NREM sleep. (**A**) Screams (in orange) and neutral vocalizations (in gray) evoke slow-waves, with maximal amplitude on Fz electrode (top panel) and less prominent on Cz (middle panel) although initial auditory-evoked potentials can be visually observed on Cz before 0.6 s. Topographies (at the bottom) of the P200, N550 and P900 are typical of evoked slow-waves during sleep, with maximal amplitude on frontal electrodes. (**B**) Power time-frequency map averaging responses across all electrodes reveals enhanced power in the delta, theta and sigma frequency ranges. (**C**) Power responses averaged in the sigma (top panel), theta (middle panel) and delta (bottom panel) frequency ranges reveal an increase in all ranges investigated for both screams and neutral vocalizations except in the sigma range where only screams induce a significant increase. Note how the power in all bands qualitatively appears to be spread out over time relative to the awake state. (see Fig. 2D) (**D**) ITPC frequency map indicates that, as during wakefulness, vocalizations evoke phase-locked responses in the delta-theta (1–8 Hz) range. (**E**) Averaged ITPC response in the theta (4–8 Hz) range are larger for screams (orange trace) than for neutral vocalizations (gray trace), which are merely detectable. Plain lines indicate the mean response across participants and shaded surfaces the SEM in plots of D and E. Significant statistical differences (corrected using cluster-based permutations) are plotted as thick horizontal lines (orange for screams against 0; gray for neutral vocalizations against 0 and black for the difference screams vs. neutral vocalizations).

The analysis of ERP during NREM sleep shows that both scream and neutral conditions evoked large deflections (slow-waves) with highest amplitude on frontal electrodes (Fig. 3A; top panel) with “earlier” P200 and N350 components on the central Cz electrode (Fig. 3A; middle and bottom panel), consistent with the existing literature^13^. The three main components of evoked slow-waves (P200, N550 and P900) typically displayed highest amplitudes on frontal electrodes. No difference between screamed and neutral vocalizations were observed in the time courses of ERPs on Fz ( No cluster was found between 0 and 1 s post-stimulus onset) or Cz electrodes (No cluster was found between 0 and 1 s post-stimulus onset).

We then extracted the power of evoked oscillations that are typical of ERPs (i.e. theta frequency range) and of NREM sleep oscillations (i.e. spindles in the sigma frequency range and slow-waves in the delta frequency range). Using the same time-frequency decomposition approach as in the wake data, we found that spectro-temporal response patterns were mostly conserved during sleep. We first observed that screamed and neutral vocalizations evoked low-frequency responses in the delta [1–4 Hz] frequency range (Fig. 3B; bottom right panel; significant cluster of the difference screams vs. baseline from 0.180 to 1.880 s; mean power difference ± SD (dB): 9.78e-01 ± 1.28, *N* = 15, t_cluster_=237.77, p = _cluster_0.004, d = 0.77; significant cluster of the difference neutral vocalizations vs. baseline from 0 to 1.860 s; mean power difference ± SD (dB): 8.60e-01 ± 1.22, *N* = 15, t_cluster_=248.52, p_cluster_=0.007, d = 0.70), as well as in the theta frequency range (Fig. 3B; middle right panel; significant cluster of the difference screamed vocalizations vs. baseline from 0.240 to 2.00 s; mean power difference ± SD (dB): 6.88e-01 ± 8.37e-01, *N* = 15, t_cluster_=256.63, p_cluster_=0.001, d = 0.82; significant cluster of the difference neutral vocalizations vs. baseline from 0.000 to 0.780 s; mean power difference ± SD (dB): 6.18e-01 ± 9.82e-01, *N* = 15, t_cluster_=95.62, p_cluster_=0.017, d = 0.63). Focusing on the theta range, we found an overall increase of auditory responses in sleep relative to wakefulness at the single-trial level in all conditions (Fig S2A; Significant cluster from 0.560 to 1.840 s; mean power difference ± SD (dB): 6.30e-01 ± 8.19e-01, *N* = 15, t_cluster_=184.89, p_cluster_=0.005, d = 0.77) and an overall decrease of ITPC (Fig S2B; significant cluster of the difference wake vs. NREM sleep from 0.040 to 0.660 s; mean ITPC difference ± SD (a.u.): 1.10e-02 ± 1.99e-02, *N* = 15, t_cluster_=-113.22, p_cluster_=0.002, d = 0.55). When splitting across auditory conditions, screamed vocalizations elicited a significant theta ITPC increase (Fig. 3C; right panel; significant cluster from 0.120 to 0.320 s; mean ITPC difference ± SD (a.u.): 1.41e-02 ± 2.19e-02, *N* = 15, t_cluster_=26.39, p_cluster_=0.035, d = 0.64), whereas neutral vocalizations did not (No cluster was found between 0 and 1s post-stimulus onset). Moreover, ITPC magnitude was significantly larger for screamed than neutral vocalizations in a similar time window [0.240 to 0.480 s] as observed during wakefulness (significant cluster from 0.120 to 0.320 s; mean ITPC difference ± SD (a.u.): 1.41e-02 ± 2.19e-02, *N* = 15, t_cluster_=26.39, p = _cluster_0.035, d = 0.64). Accordingly, when filtering individual ERPs recorded during NREM sleep within the theta range, the response to screams looks more similar across participants as compared to neutral vocalizations (Fig. S1, lower panels). As screamed vocalizations were matched paired in pitch with neutral ones (i.e., produced by the same eight actors), this result supports a specific effect of roughness. In line with our main hypothesis, this crucial acoustic feature –even at a very low sound level– enhances theta phase-consistency, potentially promoting fast and reliable processing of auditory stimuli.

In addition to an increase in theta power and slow-waves, time-frequency analyses revealed an increase in higher frequencies, between 12 and 15 Hz, a range that is typical of fast spindles^11^ (Fig. 3B; left panel). Because increased evoked sleep spindles is generally considered as a marker of stimulus salience^2,6,27^, we tested whether high-sigma power increase was affected by the type of vocalization. First, we observed that sigma power increased in response to screamed vocalizations (Significant cluster from 0.580 to 1.980 s; mean power difference ± SD (dB): 7.83e-01 ± 1.03, *N* = 15, t_cluster_=201.63, p_cluster_=0.003, d = 0.76) but not after neutral vocalizations (No cluster was found between 0 and 2s post-stimulus onset). When directly comparing responses between conditions, we found that screamed vocalizations induced larger responses in the sigma range at late latencies, between 0.700 and 1.360 s (significant cluster mean power difference ± SD (dB): 3.93e-01 ± 4.92e-01, *N* = 15, t_cluster_=93.26, p_cluster_=0.011, d = 0.80; Fig. 3B; top right panel).

### Relationship between roughness and evoked sleep oscillations

**Fig. 4:**
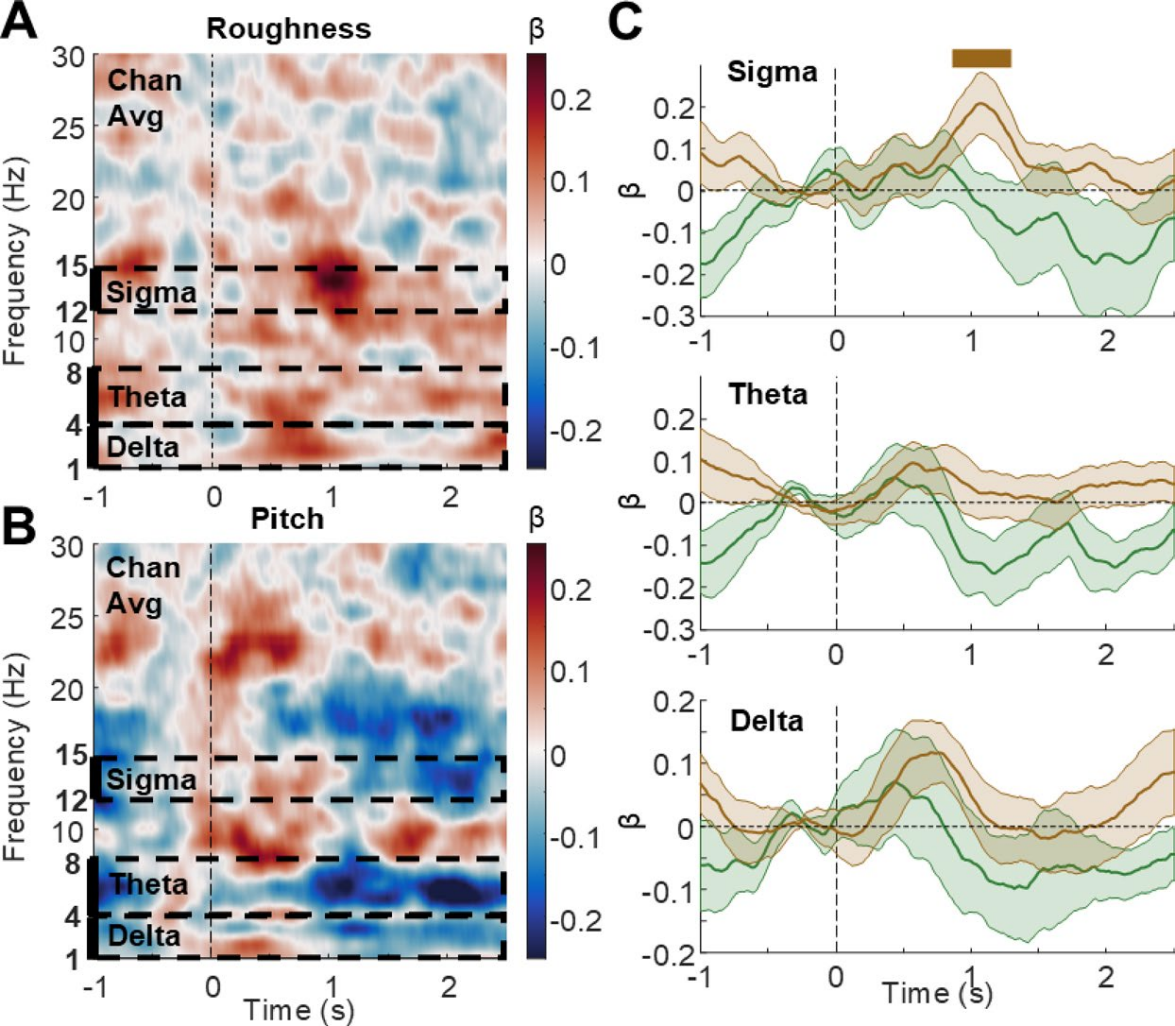
Roughness (but not pitch) enhances evoked responses during sleep. (**A**, **B**) Regression of evoked responses (at each time point and frequency) by stimulus roughness (**A**) or pitch (**B**). Black dotted rectangles indicate time-frequency windows of interest in sleep-related frequency ranges, namely in the sigma (12–15 Hz) and delta (1–4 Hz) ranges, for subsequent statistical analyses. (**C**) Time-course of sigma, theta and delta power regressions by roughness (brown) and pitch (green). While the pitch of vocalizations does not seem to influence brain responses during sleep, roughness induces a significant delta- followed by a sigma-power increase during auditory stimulation. Shaded surfaces indicate SEM.

To confirm that roughness specifically underlies the differential response to screamed and neutral vocalizations in NREM sleep, we assessed how much roughness and pitch levels affected the generation of slow-waves and spindles, two typical spectral signatures of exogenous processing during sleep. To this end, we used regression analyses between vocalizations’ roughness or pitch and the power of frequency-resolved responses. As expected from our previous observations, a qualitative inspection of these maps suggested that roughness (Fig. 4A), unlike pitch (Fig. 4B), enhanced the power of brain responses in the sigma frequency range (dashed black rectangles), consistent with an increased generation of spindles by roughness,. Using a single-trial regression analysis approach, we assessed the relationship between the acoustic features of interest (roughness and pitch) and specific frequency-bands of spindles across time in the sigma (12–15 Hz; Fig. 4C) ranges. We found a significant relationship between roughness and sigma power in a later 0.860 to 1.300 s time-window (Significant cluster with mean β ± SD: 1.73e-01 ± 2.41e-01, *N* = 15, t_cluster_=60.12, p_cluster_=0.022, d = 0.72; Fig. 4C), whereas no significant power gain was observed in the regression with pitch values (No cluster was found between 0 and 2 s post-stimulus onset).

Using the same approach, we observed no changes in the delta (Marginal cluster in relation to roughness from 0.640 to 0.880 s; mean β ± SD (dB): 1.11e-01 ± 1.78e-01, *N* = 15, t = 30.96, *p* = 0.066, d = 0.62 and no cluster was found in relation to pitch) and theta (No cluster was found in relation to roughness and the cluster in relation with pitch with the lowest p-value was found from 1.100 to 1.180 s; mean β ± SD (dB): -1.58e-01 ± 2.74e-01, *N* = 15, t=-11.11, *p* = 0.122, d = 0.58).

## Discussion

A large body of experimental evidence supports the idea that the brain maintains some level of responsiveness to auditory stimuli during sleep^1–9,28,29^. However, it remains unclear whether the brain prioritizes certain acoustic features of sounds over others, and how it maintains an acoustic sensitivity to emotionally-relevant sounds during sleep. Sound loudness, which indexes the spatial distance and physical size of the source, is perhaps the most relevant feature to indicate an imminent threat in the environment. Yet, because intensity attenuates with distance, this feature can only ambiguously inform about the urgency of an alarm call. Unlike intensity, roughness is selectively used as a warning signal. As such, it provides unequivocal cues regarding the communicative intent of the emitter (i.e., to warn conspecifics) and may trigger neural and behavioral responses even when present at low intensity. Here, we hypothesized that roughness, as an innate and universal acoustic feature for signaling danger, might be processed in a privileged manner during sleep. We thus hypothesized that, owing to their aversiveness and their propensity to capture attention, rough sounds (as featured in human screams but also in wake-up devices such as alarm clocks and buzzers) are likely to be processed more efficiently than smoother (less rough) neutral sounds during sleep^15,16^. We further assumed that varying roughness levels (but not necessarily pitch) should be reflected in typical electrophysiological markers of sensory processing during sleep.

### Roughness enhances exogenous neural synchronization during NREM sleep

Here, we show that, even at a low intensity, screams induced higher phase-consistency across responses in the theta range than neutral vocalizations in both wakefulness and NREM sleep. This effect manifested as increased ITPC of early evoked responses in the theta frequency range which is outside the influence of evoked slow-waves. Although the ITPC metric can be influenced by the power of the signal^30^, no difference in power and no difference of ERP amplitude (which is influenced by both power and ITPC metrics of oscillations) was observed between the two types of stimuli. Consequently, such specific ITPC increase can be interpreted as indicating that roughness induced more reliable and stereotyped stimulus-driven neural response. This effect is in line with other studies linking auditory sensory salience to enhanced response consistency across trials in wakefulness^18,19^ and sleep^17^. Similarly, roughness in screams might exogenously boost the synchrony of activity of neuronal populations across emotional and auditory areas, thus increasing the phase-consistency and promoting rapid and more efficient detection of salient sounds. This is consistent with the findings that rough sounds enhance neural synchronization throughout temporal cortical and subcortical limbic networks involved in salience and emotional processing^16^. In this study, whereas evoked responses varied linearly with increasing stimulus train rates (10 to 250 Hz) in the auditory cortex, sounds in the roughness (~ 30–80 Hz) range additionally enhanced exogenous synchronization in neuronal networks involved in arousal and salience processing. This observation nicely aligns with our result that naturally rough vocalizations (screams) produce more consistent brain responses on a trial-by-trial basis. Importantly, the current findings further demonstrate that this sensory coding property extends to another vigilance state, sleep. This is also consistent with other works investigating brain responses to 40-Hz click trains during sleep. This rough artificial sound entrains cortical response at the same frequency (40 Hz in this case) in wakefulness, a phenomenon called auditory steady-state response (ASSR). These studies converge to show that ASSR is only slightly dampened during NREM sleep^29,31,32^ and thus, suggest that the sleeping brain is still sensitive to acoustic roughness (although the ASSR was not compared with slower, less rough, click train rates in that case).

### Neural routing of emotionally inductive signals

Whether and how low-level features such as roughness can specifically target brain networks involved in attentional and affective processing remains a matter of active research^15,16,33^. In the visual domain, emotional stimuli, like angry or happy faces, can boost the activity of neural populations in emotional (e.g. amygdala) and sensory cortices^34,35^. In turn, emotional items are detected faster than neutral items^36,37^. Mirroring these results, in the auditory domain, some features of emotional prosody of meaningless utterances such as laughs, cries, and screams, lead to higher metabolic activity of the amygdala and enhanced auditory processing^15,38–41^. Among these features, roughness appears to mediate amygdala responses to screams^15^ but the neural routing of such stimuli remains unclear. In line with the direct route hypothesis, these sounds may be directly addressed to emotional centers of the brain via rapid direct pathways^42^. In this view, roughness would potentiate the processing of alarm vocalization (and potentially of other emotional vocalizations) by rapidly and exogenously entraining neuronal synchrony in emotion-related brain regions and attentional networks.

In line with recent evidence, the current results support the hypothesis that rough sounds promote aversion by triggering sustained neuronal synchronization across widespread brain networks^16^. The spatio-temporal profile of such responses described in previous publications is compatible with the recruitment of non-canonical auditory pathways ultimately targeting salience processing systems^16,33^. The precise neural routes underlying such extensive synchronization by rough sounds remain unclear. Because roughness processing appears preserved during sleep, this could also point towards a potential implication of deep cerebral circuits involved in sleep regulation, as suggested by recent animal work^43,44^. Specifically, by targeting and synchronizing such subcortical systems during sleep, rough sounds would efficiently allow the cerebral cortex to process and react to signaled danger.

The larger evoked slow-waves and spindles in response to rougher vocalizations are also consistent with a potential involvement of arousal-promoting neuromodulatory groups of the brainstem^45,46^. Sensory pathways projecting through the brainstem, such as lemniscal and non-lemniscal routes, follow distinct circuits. Non-lemniscal nuclei, in particular, engage in bidirectional communication with a broad network of midbrain, cortical, and limbic regions. These connections provide channels for auditory information to reach higher cortical areas, facilitate transitions between arousal states, and synchronize activity across extensive cortical territories^47^. Notably, sleep spindles naturally occur more frequently in brain regions involved in attention and emotion regulation^48–50^ – such as the dorsolateral prefrontal cortex, the cingulate cortex, the orbitofrontal cortex, motor areas, and the precuneus – which are also regions of high noradrenaline and acetylcholine receptor density^51^. Since roughness affects the phase consistency –but not the power– of brain responses, this may suggest that such stimuli may modulate sensory processing via a phase-resetting mechanism, rather than through classical feed-forward drive along the classical auditory pathway^52^. In this framework, rough sounds could trigger the exogenous recruitment of non-lemniscal (cholinergic) pathways, which may explain the widespread cortical synchronization observed and the enhancement of perceived salience^16^. The increase of slow waves and spindles could also reflect the recruitment of additional sensory or non-sensory thalamic structures by rough sounds^11,53^. These oscillatory signals, typically generated through interactions between the thalamic reticular nucleus (TRN), thalamocortical neurons, and cortical feedback, are critical for sleep stabilization^54–56^, aligning with the idea that the processing of such sounds during sleep require counteracting sleep mechanisms to prevent awakenings (see below). In line with these notions, alterations in the thalamus and brainstem in some comatose patients may lead to reduced brain responses to sounds in the roughness range^57^. However, although fascinating, the idea that rough sounds would selectively recruit such pathways still needs further anatomical and functional evidence from animal models.

### Acoustic roughness boosts sleep oscillatory activity

By stimulating participants with rough and smooth vocalizations during sleep, we showed that rough vocalizations (i.e., screams) yielded a transient increase in sigma power, as compared to smooth vocalizations. In addition, we observed that the rougher the vocalization, the stronger the evoked sigma power. Sleep spindles, which are the main source of sigma power during NREM sleep, are linked to sleep maintenance, as they are more abundant for people with stable sleep^54^ and during periods of sleep more resistant to disturbances^58^. In addition, sleep spindles can be triggered by external stimuli^27^ and are potentiated by stimulus salience^3,5,6^ (but see also Ameen et al., 2022^7^). Hence, some authors proposed that evoked slow-waves and sleep spindles represent intermediate responses on the arousal spectrum between small brain responses (e.g., ERPs) and micro-arousals or awakening^6,55,59^. Thus, whenever a stimulus might be assigned to a moderate threat (in our case, the low intensity might dampen the threatening aspect of screamed vocalizations), yet salient enough, it is likely to enhance sleep oscillations across broad networks, while full arousal would not be necessary. To ensure sleep stability, spindle generation might prevent further processing of sensory stimulation. Vocalizations’ roughness also increased early delta power, a marker of slow-wave activity. Much like what was reported above for spindles, the generation of slow-waves appears to be linked to stimulus salience^13^. Thus, as rough vocalizations evoked more slow-waves and spindles in the present study, we argue that roughness, even when presented at very low intensity, constitutes an efficient attribute to mobilize exogenous attentional systems during wakefulness as well as during sleep. ITPC analysis revealed that the phase-alignment of brain responses to screams was more consistent across trials, which suggests a more reliable processing of auditory information and which could lead to a more systematic generation of sleep spindles and slow-waves by rougher vocalizations.

Light (N2) NREM sleep is often more sensitive to disturbances than deep (N3) NREM sleep and is denser in spindles too. Such spindle response in face of salient vocalizations may be boosted in N2 sleep but distinguishing N2 from N3 was beyond the scope of this study. More studies need to be carried out to reveal whether responses to rough sounds differ between N2 and N3 or between fragile and stables phases of N2 for example. The precise mechanisms by which acoustic roughness influences the sensory processing of sounds remain unclear. Previous research has linked acoustic roughness with aversiveness^15,16^ and salience^60^, and we have proposed a neurobiologically grounded explanation (see section above, *Neural Routing of Emotionally Inductive Signals*). However, whether acoustic roughness also enhances other perceived sound qualities – such as perceived loudness – which mediate these effects requires further investigation and was beyond the scope of this study.

The direct comparison between the evoked responses to screams and neutral vocalizations in NREM sleep highlights two crucial findings: (1) roughness is a salient acoustic feature irrespective of vigilance state and orthogonal to sound intensity, and (2) sigma power is a reliable marker of roughness-related stimulus salience in NREM sleep. These results suggest that roughness remains efficiently processed even when presented at very low intensity during sleep. By triggering reliable responses in a state of reversible unresponsiveness such as NREM sleep, roughness constitutes an adaptive communication channel, which would ensure that warning signals such as screams can be optimally processed from a distance by sleeping conspecifics. The current findings might also account for the aversiveness of rough snoring sounds and their detrimental effects on sleep^61^. Many human activities generate rough sounds^15,62^, which might disturb sleep by enhancing the recruitment of arousal-related brain circuits^63,64^. These results thus offer strong scientific incentive for the careful consideration of specific sound attributes, such as roughness (and beyond mere sound intensity), when promoting healthier sleeping environments.

## Electronic supplementary material

Below is the link to the electronic supplementary material.

Supplementary Material 1

## Data Availability

EEG data supporting this study are freely available at the following link: 10.5281/zenodo.8407715. Audio files of the auditory stimuli used in this study are available at the following link: 10.5281/zenodo.14267509.

## Code availability

Custom scripts are available upon reasonable request.
