# Supplementary material for: Scream’s roughness grants privileged access to the brain during sleep
Source: Sci Rep. 2025 May 14;15:16686. doi: 10.1038/s41598-025-01560-8 (PMC12078618; doi:10.1038/s41598-025-01560-8)
Supplement: Supplementary file 1 — Supplementary Material 1 [file 41598_2025_1560_MOESM1_ESM.pdf]

Supplementaries

Scream’s roughness grants privileged access to the brain during sleep.

Guillaume Y.T. Legendre, Maëva Moyne, Judith Domínguez-Borràs, Samika Kumar, Virginie Sterpenich, Sophie Schwartz, Luc H. Arnal

Corresponding author: Guillaume Y.T. Legendre
Email: guillaumeyt.legendre@gmail.com

This PDF file includes:

Figures S1
Figures S2

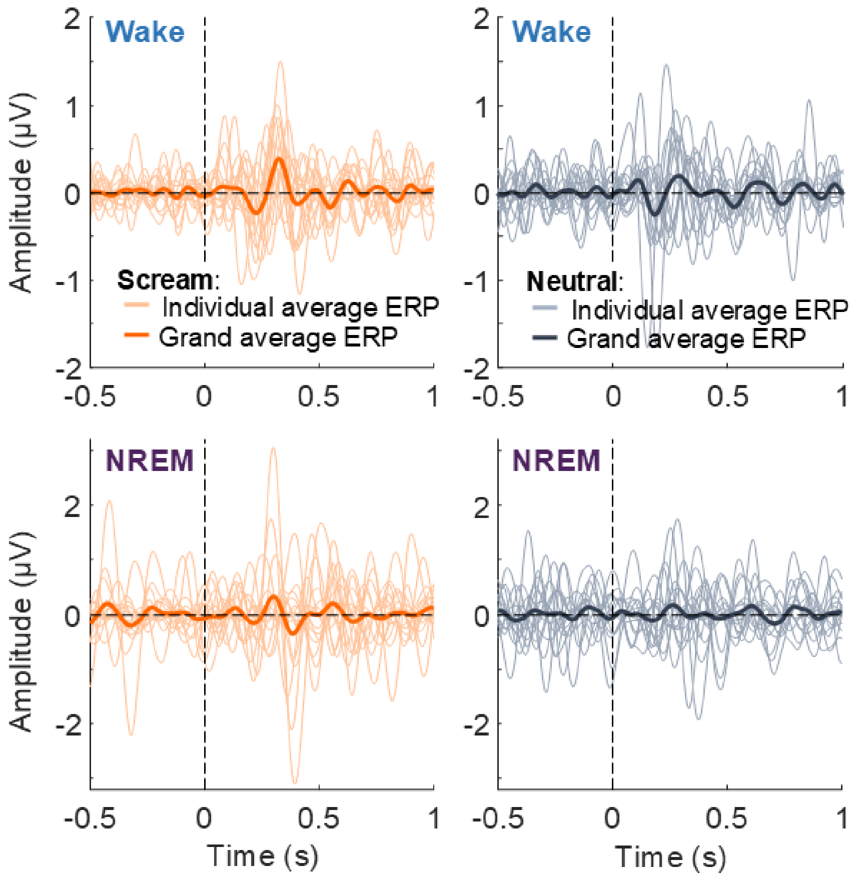

Fig. S1. Individual and grand average ERPs filtered in the theta (4-8 Hz) frequency band. The plots depict the average brain response recorded on Cz of each participant (light traces) and the grand average across participants (bold trace) in response to screams (orange; left panels) or in response to neutral vocalizations (grayish blue; right panel). Upper panels show the response recorded at wakefulness and lower panels the responses recorded during NREM sleep.

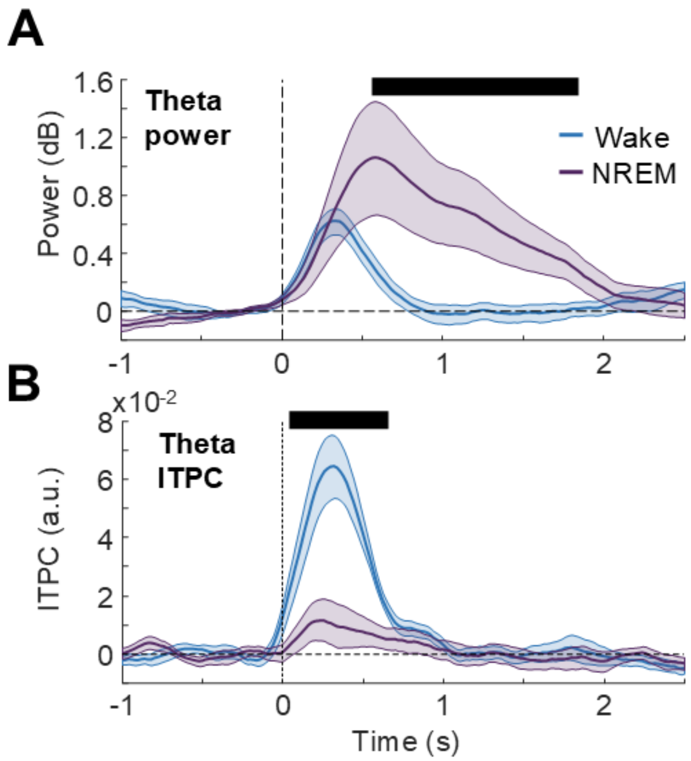

Fig. S2. A,B. Comparison of power (**A**) and ITPC (**B**) of brain responses at wakefulness (blue) and in NREM (dark purple) in the theta frequency range to screams and neutral vocalizations together. Plain lines represent the means across participants and shaded area the SEM. Thick horizontal lines represent significant differences of response between wakefulness and sleep.
